# Saccharides as Particulate Matter Tracers of Biomass Burning: A Review

**DOI:** 10.3390/ijerph19074387

**Published:** 2022-04-06

**Authors:** Beatrice Vincenti, Enrico Paris, Monica Carnevale, Adriano Palma, Ettore Guerriero, Domenico Borello, Valerio Paolini, Francesco Gallucci

**Affiliations:** 1 Council for Agricultural Research and Economics (CREA), Center of Engineering and Agro-Food Processing, Via della Pascolare 16, 00015 Monterotondo, Italy; beatrice.vincenti@crea.gov.it (B.V.); enrico.paris@crea.gov.it (E.P.); monica.carnevale@crea.gov.it (M.C.); francesco.gallucci@crea.gov.it (F.G.); 2 National Research Council of Italy, Institute of Atmospheric Pollution Research (CNR-IIA), Via Salaria km 29,300, 00015 Monterotondo, Italy; guerriero@iia.cnr.it (E.G.); valerio.paolini@iia.cnr.it (V.P.); 3 Department of Mechanical and Aerospace Engineering, Sapienza University of Rome, Via Eudossiana 18, 00184 Rome, Italy; domenico.borello@uniroma1.it

**Keywords:** biomass burning, saccharides, anhydrosugars, sugar alcohols, tracers, PM

## Abstract

The adverse effects of atmospheric particulate matter (PM) on health and ecosystems, as well as on meteorology and climate change, are well known to the scientific community. It is therefore undeniable that a good understanding of the sources of PM is crucial for effective control of emissions and to protect public health. One of the major contributions to atmospheric PM is biomass burning, a practice used both in agriculture and home heating, which can be traced and identified by analyzing sugars emitted from the combustion of cellulose and hemicellulose that make up biomass. In this review comparing almost 200 selected articles, we highlight the most recent studies that broaden such category of tracers, covering research publications on residential wood combustions, open-fire or combustion chamber burnings and ambient PM in different regions of Asia, America and Europe. The purpose of the present work is to collect data in the literature that indicate a direct correspondence between biomass burning and saccharides emitted into the atmosphere with regard to distinguishing common sugars attributed to biomass burning from those that have co-causes of issue. In this paper, we provide a list of 24 compounds, including those most commonly recognized as biomass burning tracers (i.e., levoglucosan, mannosan and galactosan), from which it emerges that monosaccharide anhydrides, sugar alcohols and primary sugars have been widely reported as organic tracers for biomass combustion, although it has also been shown that emissions of these compounds depend not only on combustion characteristics and equipment but also on fuel type, combustion quality and weather conditions. Although it appears that it is currently not possible to define a single compound as a universal indicator of biomass combustion, this review provides a valuable tool for the collection of information in the literature and identifies analytes that can lead to the determination of patterns for the distribution between PM generated by biomass combustion.

## 1. Introduction

Any solid or liquid fuel combustion event generates airborne material that negatively affects air quality and health. This is due to the fact that volatile and unburnt products are inevitably generated by combustion processes that cannot in any way be ideal [1,2]. Such combustion events are the major sources of aerosols and exert significance influence on human health, air quality and global climate [3,4].

The term “aerosol” designates the complex of solid and liquid particles suspended in the atmosphere that may vary in size from a few nanometers to tens of microns [5]. Depending on their origin, aerosols may be natural or anthropogenic and may consist of mixtures of organic and inorganic compounds, primarily emitted or formed in the atmosphere from precursor gases (secondary aerosols), exhibiting a broad and complex spectrum of physical and chemical properties, which actively contribute to their climate and health-related effects [4,6]. Organic aerosols are the essential components of airborne particulate matter (PM), globally recognized as one of the main environmental and health risk factors contributing to the development or exacerbation of many diseases [7].

Epidemiological studies have found strong relationships between cardiovascular outcomes, respiratory illnesses and asthma and the high level of organic aerosols in the atmosphere [8]. Studies on pollution levels provided evidence concerning premature mortality in Europe, which totals at least 40,000 premature deaths per year [9]. Several experimental studies attribute the onset of adverse health effects, such as eye and throat irritation, to wood smoke, and inhalation studies have demonstrated that wood smoke exposure may induce systemic effects, providing a possible link to cardiovascular effects [9,10].

Evidence from a large number of studies shows that organic aerosols affect not only human health but also influence climate. They have become a topic of great interest because of their influence on radiative forcing, biogeochemical cycles and atmospheric chemistry [11,12,13].

Jimenez et al., 2009 [14] stated that organic aerosol (OA) can modify the earth’s climate through scattering and absorption of solar radiation, altering cloud properties and lifetime, whereas Cao et al., 2012 [15] considered the ability of particles to reduce visibility by causing uptake of water into the aerosol.

Accounting for 18–38% of the fine OA [16], biomass burning significantly contributes to the emission of gases and toxic compounds in the atmosphere [17,18], and it has been identified as a biofuel that may contribute to the worsening of both outdoor and indoor air quality [19]. In central and northern Europe, biomass burning emissions have been recognized as one of the major sources of organic aerosols during wintertime, contributing, as domestic heating, to air quality degradation in many Mediterranean urban areas [20,21,22]. In addition to biomass burning for residential heating, burning of agricultural waste is a common practice that emits relevant amounts of gaseous and particulate pollutants into the atmosphere [23,24]. In developing countries, although environmentally unacceptable, *open burning* is a widespread practice used as a rapid, cheap and easy method for disposing of crop residues, releasing nutrients for the next growing cycle and clearing lands [25].

Nowadays, renewable energy sources in the form of solid biomass are becoming increasingly important in order to replace fossil fuels and reduce greenhouse gas emissions [26,27]. Solid waste biomass may be an alternative to conventional energy sources, mostly in the form of compressed biofuels; briquettes or pellets have the advantages of high density, lower moisture and better physical homogenization. There are many specific papers focused on the characterization, production and energy evaluation of such biomass materials as fuels [28,29], which represent an important opportunity in the renewable energy field.

This study seeks to collect and define the information available in the literature on the set of biomarkers that contribute to the PM produced both by natural phenomena and by conversion processes of energy from biomass in order to be able to differentiate the PM emitted by natural or anthropogenic activities (such as the use of fossil fuels).

In this review, biomass burning is observed in the form of open burning of agricultural residues, grassland and forest fires, and residential combustion of biomass for cooking and heating purposes. In this context, the most widespread biofuels include both crop waste, such as rice straw, maize residue, wheat residues and bean straw [30]; and woody fuels, such as branches and wood [31].

Although in the literature, there are several studies aimed at determining sugar compounds present in PM emitted from anthropogenic or natural sources [32,33,34,35,36] and there are also numerous studies based on the source apportionment of such aerosols [37,38,39,40,41,42], to the best of our knowledge, there is a lack of studies related to determination and quantification of such compounds as possible tracers useful for the discrimination of PM emitted by biomass combustion. The purpose of this review differs from that of the aforementioned papers, bringing together multiple results concerning biomass-derived saccharides.

## 2. Methodology

**Figure 1:**
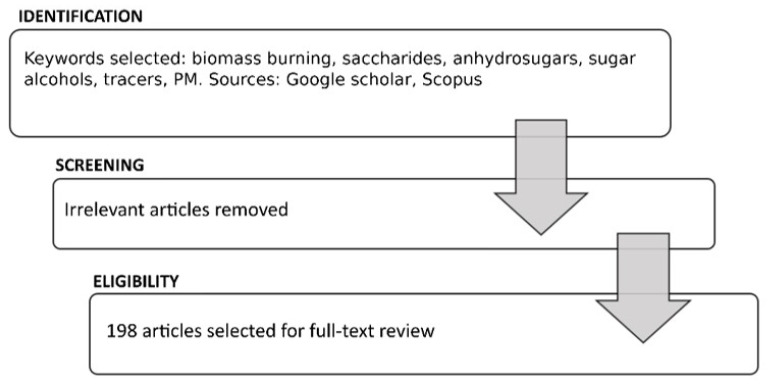
Flowchart describing the literature research and article selection.

A collection of data in the global scientific literature was conducted to determine which saccharide compounds have been observed and quantified during atmospheric monitoring in relation to biomass combustion phenomena and those attributed to natural causes. This was achieved through an extensive literature research (Figure 1) performed of the SCOPUS and the Google Scholar databases, using the keywords: biomass burning, tracers, saccharides, anhydrosugars, sugar alcohols and PM. A total of 198 scientific papers published during the period of 1988–2022 were considered, and it was possible to organize the review work into several sections. Section 3 deals with the characterization of saccharides identified as tracers during environmental monitoring campaigns and summarizes the major source apportionment studies aimed at the identification of such marker compounds; Section 4 presents the results of such identification, and Section 5 provides a summary and concluding remarks for future research directions. Reviewing the extensive literature on aerosol emissions from biomass combustion, in this paper, we aim to provide an overview of the contributions of sugar compounds to PM, synthesizing results from published literature and summarizing the factors governing such emissions.

## 3. Biomass Burning

Biomass is rich in saccharides in the form of oligosaccharides and polysaccharides, such as cellulose, lignin and starch, which, upon combustion, produce significant amounts of monosaccharides, disaccharides, sugar alcohols and anhydrosugars, in addition to other simple molecules [43]. Such saccharide compounds are one of the major classes often utilized as biological markers for atmospheric aerosols [44,45,46], and by virtue of their ubiquity and size-resolved chemical composition, they have offered the opportunity to estimate the atmospheric trajectory and sources of aerosol particles [47,48,49]. The anhydro saccharides levoglucosan (L), mannosan (M) and galactosan (G) originate from the combustion of cellulose and hemicellulose and are accordingly recognized as biomass-burning markers [50,51,52]. Primary saccharides such as glucose, sucrose and fructose are characteristic of material such as pollen, fruit, and plant fragments [53], whereas sugar alcohols, such as mannitol and arabitol are characteristic of fungal spores [54]. However, several studies have attributed biomass burning as the source of these last two categories of saccharides, finding possible causes of increased concentrations of such saccharide compounds in the atmosphere in the volatilization from breakdown of polysaccharides and in hydrolysis in conditions of atmospheric acidity during burning [55,56,57,58].

Table S1 shows a list of such saccharide compounds monitored and emitted from natural or anthropogenic sources, with their respective concentrations, locations, PM fractions, technique, background and period of monitoring. Not all studies have as their main objective the quantification of the identified compounds, so some studies, such as [59] and [60], do not present the concentrations of the identified saccharides.

Characterizing compounds at a particle scale can thus improve our knowledge of the composition of aerosols from combustion-related emission [36]. Studies have focused on the seasonal and temporal variations of atmospheric sugars in several areas throughout the world [61,62,63,64]. Research in this field is crucial because it provides useful data regarding the sources and processing of aerosols released into the atmosphere from biomass burning, soil dust and primary biological aerosols, such as fungal spores and pollen, all of which have a considerable impact on the environment.

### 3.1. Tracers

As mentioned above, biomass burning is an important primary emitter of several trace organic compounds that are reactive in the atmosphere, as well as of soot particles, which decrease visibility and absorb incident radiation [65,66]. For this reason, the composition of atmospheric aerosols has received increasing attention, aimed at determining the contributions of the various emission sources to environmental PM. Khalil and Rasmussen, 2003 [67] argued that a tracer may be considered ideal when certain peculiar characteristics are met: it has to be resistant to degradation, source specific and constant and allow for high-precision measurement. However, some factors, such as the complex chemical combustion conditions, the high inhomogeneity of the particles, the different types of biofuel used, the operating conditions or the formation of ash, can make it difficult to find a tracer that meets all of these characteristics at once [68,69,70,71,72,73,74]. Water-soluble potassium (K^+^) has been proposed as a tracer for biomass burning in receptor models due to its ubiquity in the cytoplasm of plants [75], and a strong positive correlation was observed among K^+^, organic carbon (OC), elemental carbon (EC) and WSOCs (water-soluble organic compounds) that indicated crop residues burning to carbonaceous aerosols [76]. Several studies in the literature have also proposed the use of the levoglucosan to K^+^ ratio to distinguish the particular plant species of BB activities in atmospheric aerosols [77]. However, fertilizers and soil dust resuspension in rural areas [18,78,79], as well as meat cooking and refuse incineration in urban areas [80,81,82], produce and release K^+^ in the atmosphere, leading to many limitations in the use of this marker.

Factors such as varying temperature conditions, aeration, heating temperature, and smoldering and flaming conditions contribute to determining the nature and amounts of the combustion products [83]. Due to their source-specific origins, some thermally altered molecules can therefore be used as chemical fingerprints and be useful in determining the contributions of the burning of different biomasses to atmospheric particulate matter.

Research conducted in recent years indicates that anhydrosugars can be produced and emitted into the atmosphere by the burning of coals, especially lignites [84,85]. This is noteworthy, considering that in some countries, such as Poland and China, emissions of carbonaceous PM are highly connected with coal burning [86,87,88], and this inevitably associates the emission of a portion of detected anhydrosugars with such coal fuels. Admittedly, this is not the issue that this work is concerned with, and deeper insights are provided in the literature [51,89,90].

### 3.2. Source Apportionment Studies

Air quality is strongly affected by PM emissions generated by multiple sources, such as industrial processes, vehicular traffic, power plants, combustion of agricultural and food residues, and uncontrolled forest fires [91,92]. Accurate analysis of pollutant sources and their components is a crucial step toward developing efficient control strategies and reducing the harmful effects of particulate matter [93,94,95]. Among the developed methods, the receptor model is a widely used tool for PM source apportionment (SA) studies. Such methods can be categorized into univariate models, e.g., chemical mass balance (CMB), and multivariate models, such as positive matrix factorization (PMF), principal component analysis (PCA) and the EPA’s Unmix model [96,97]. However, the CMB model has limited application because it requires prior knowledge about source profiles and, in order to achieve quantitative SA, needs to be associated to other methods. On the other hand, the Unmix model does permit quantification of the source contribution, although it cannot separate sources that have similar contributions to particle mass [98]. In contrast, PMF is a multivariate chemical receptor model based on factor analysis developed by Paatero at the University of Helsinki, Finland [99,100]. Briefly, this receptor model can estimate the factor profiles and corresponding relative contributions during the sampling period based on a large amounts of observation data, overcoming the aforementioned problems using a least-square method to assess the source profiles and their contributions to particle mass [101,102].

Further explanation of receptor models, their assumptions and applications can be found in [103,104,105,106].

In the context of the literature studies we reviewed, many have used these SA methods to identify and quantify the contribution of biomass burning to total atmospheric PM, allowing for attribution of the presence of saccharides in the air to both biogenic and anthropogenic factors, such as biomass burning. Table 1 shows the main source apportionment studies that have attributed sugars to biomass burning and natural sources. As can be seen, the most commonly used SA technique is PMF, followed by PCA.

Thus, levoglucosan and its isomers are used as specific chemical markers to identify biomass-burning-derived emissions and help in source apportionment approaches. Such compounds have been used in studies around the world: in Europe [84,107,108,109,110], North and South America [111,112,113], as well as Asia [114,115,116,117,118,119].

Additionally, sugar alcohols such as inositol and arabitol have been proposed as biomass-burning-derived tracers. Originated from the metabolism of fungi and found on leaves of trees, such carbohydrates are also emitted from open-air combustion [120], stoves [121] and combustion chambers [122]. Thus, concentrations of sugar alcohols, in combination with high levels of levoglucosan, indicate that biomass combustion contributes to the aerosol content, resulting in their classification as biomass burning markers.

## 4. Saccharides

Sugar compounds have been proposed both as tracers for determining the sources, processes and paths of aerosols emitted by biomass burning and for elucidating the atmospheric level of naturally emitted aerosols [131,135,136].

**Figure 2:**
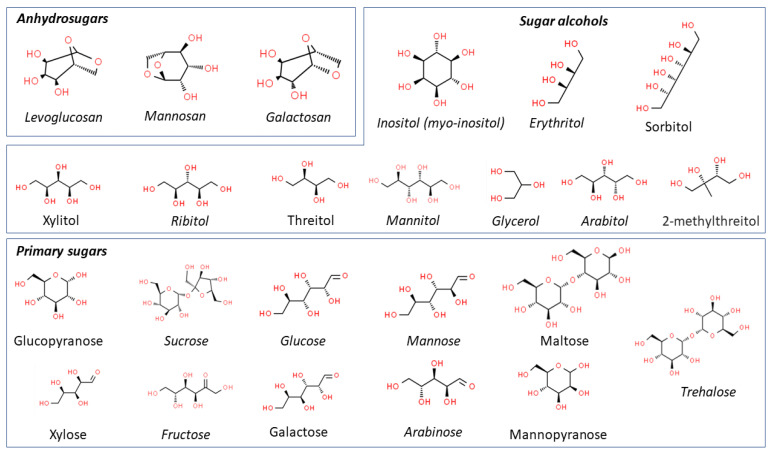
Chemical structures of saccharide compounds detected in atmospheric monitoring studies.

The organic matter in biomass is constituted of a large amount of biopolymers, such as cellulose, hemicellulose and lignin. Cellulose, providing a supporting fibrous mesh reinforced by lignin, is a long-chain linear polymer made up of 7000–12,000 D-glucose monomers able to organize to form parallel fiber structures [137], whereas hemicellulose consists of only about 100–200 sugar monomers, such as glucose, mannose, galactose and xylose, and a less intricate structure. Lignin is a coniferyl- and sinapyl-derived *p*-coumaryl and contains tannins and terpenes, which makes it a complex substance [19,138]. The biomass combustion process, which involves hydrolization, oxidation, dehydration and pyrolization phases, leads to the formation and emission of important classes of sugars, which are therefore source-specific [139,140] because, although they are also emitted from other sources (e.g., lignite or low-grade coal), the contribution of such sources can be considered negligible due to their extremely low emission rates [83,141] (Figure 2).

The main class of sugar compounds emitted by biomass combustion is anhydrosugars; in particular, levoglucosan, together with its mannosan and galactosan monomers, is the most commonly used tracer for the assessment of PM from biomass burning. Moreover, the ratio between levoglucosan and mannosan has been used to distinguish the contributions of hard and softwood to wood-combustion-related PM [142].

Sugar alcohols, such as arabitol, mannitol and inositol, are another class of sugars present in the atmosphere but emitted by biogenic sources, such as metabolism of fungi [143,144]. Many studies have focused on the existing correlation of these compounds with the anhydrosugars emitted by burning, showing that the presence of sugar alcohols, in combination with high-levels of levoglucosan and its isomer, specifically indicates that biomass burning has contributed to the aerosol content [61,145].

Mono- and disaccharides from soils and associated biota can be emitted in the atmosphere through resuspension, erosion and agricultural activities [35,146,147]. Glucose, fructose and sucrose may derive from plant pollen and developing leaves [63,131]. In light of the relevant presence of such compounds in airborne particulate matter, a comprehensive field study on the molecular and seasonal variation of atmospheric saccharides may improve our understanding of the biogenic origins of aerosol particles besides the anthropogenic sources [148].

With regard to the detection of saccharides in the monitoring campaigns that make up this review paper, the particulate fraction most investigated and in which all the categories of sugars are placed to a greater extent is PM_2.5_, followed by PM_10_ fraction and, finally, the fraction that includes particles with an aerodynamic diameter lower than 1 µm (PM_1_) [58,149].

**Figure 3:**
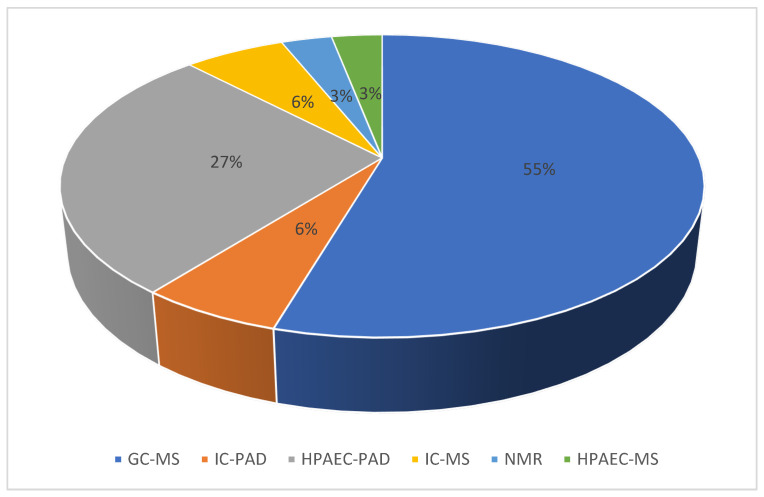
Most utilized techniques found in the literature for sugar compound determination in PM aerosol analysis (based on data from Supplementary Materials Table S1).

As far as the improvement of our knowledge is concerned, increasing scientific efforts have been demonstrated in the quantification of sugars. The most widespread analytical method for analysis of such compounds in atmospheric samples is the gas chromatographic technique coupled with mass spectrometry (GC-MS) [52,144,150,151], which guarantees high selectivity and specificity offered by the capillary columns and the m/z values in the mass spectra, respectively [152]. Limitations to such a chromatographic approach include the need for large sample mass and sample workup (e.g., solvent extraction, extract concentration and derivatization). As an alternative, liquid chromatography techniques, in the form of HPAEC-PAD (high-performance anion-exchange chromatography coupled with pulsed amperometric detection), HPAEC-MS (high-performance anion-exchange chromatography coupled with mass spectrometry), IC-PAD (ion chromatography with pulsed amperometric detection) and IC-MS (ion chromatography coupled with mass spectrometry) have been widely used to directly analyze organic compounds in aqueous filter extracts, allowing for simultaneous analysis of different sugar compounds [153,154]. An overview of the most widely used techniques revealed in this work is shown in Figure 3.

### 4.1. Anhydrosugars

Among the several categories of chemical compounds emitted by the combustion of biomass, anhydrosugars are the most frequently identified.

Levoglucosan (*1,6-anhydro-β-D-glucopyranose*) and its isomers, mannosan (*1,6-anhydro-β-D-mannopyranose*) and galactosan (*1,6-anhydro-β-D-galactopyranose*), are the most used organic tracers to assess the contribution of biomass burning to atmospheric particulate matter because, due to their low vapor pressure, they are easily found in the atmosphere as a consequence of the pyrolysis and the thermal breakdown of cellulose [10,155,156,157,158]. The presence of anhydrosugars in the ambient air has been demonstrated in emissions from prescribed and agricultural fires [44,159,160], as well as from residential wood combustion and wildfires [121,161,162].

The atmospheric concentrations of levoglucosan have shown a high variability depending not only on the type of fuel and appliance utilized but also on meteorological parameters. In a one-year PM monitoring study, Oduber et al., 2021 [161] stated that lower autumn temperatures favored an increase in the concentrations of anhydrosugars due to the increasing use of domestic heating devices. Moreover, mannosan was correlated not only with OC, EC, Pb and other heavy metals, which are fossil fuel and traffic combustion markers, but it was also correlated with As, a coal combustion marker. This result led the authors to conclude that the selection of such anhydrosugars as biomass burning tracers during the cold season may overestimate the contribution of that source because of other anthropogenic emission factors. Thus, among the numerous studies present in the literature, some cast doubt on the possibility of using anhydrosugars, particularly levoglucosan, as biomass burning tracers. Following controlled combustion experiments, some authors [163,164] came to the conclusion that the levoglucosan fraction relative to particle mass may be highly dependent on combustions parameters and that a variable that strongly influences the yield of levoglucosan is the presence of inorganic ions in the biomass [165,166], as the amount of mineral matter reduces the temperature of cellulose pyrolysis [167]. Hence, qualitative and quantitative estimates of biomass burning emissions to atmospheric PM using only levoglucosan as a marker may be unfounded.

As previously mentioned, galactosan and mannosan also contribute to biomass burning emissions, and their concentrations are strongly dependent on the biomass burned (e.g., hardwood or softwood). Based on the results available in the literature, in Table 2, the main sugars detected in different matrices burned by means of different appliances are reported.

In light of this, the simultaneous quantification of anhydrosugars is desirable in order to assess the contribution from distinct biomass burning emissions. Hence, the relative proportions of levoglucosan to mannosan (L/M) have been used for source reconstruction of combustion-derived byproducts in atmospheric aerosols. Differences in the L/M ratio in smoke from softwood and hardwood grass combustion further support discrimination between inputs from these combustion sources to the atmosphere [50]. Relatively high L/M ratios in the range of 25 to 50 can be produced by herbaceous tissues [168]. Schmidl et al. 2011 [168] investigated two automatically and two manually fired appliances, as well as eight biofuels. Detectable amounts of anhydrosugars are only emitted in the start-up phase of automatically fired systems. L/M ratios of around 14–17 for hardwoods and of 2.5–3.5 for softwood combustion using manually fired appliances were found, whereas during combustion in a biomass boiler, the authors reported L/M ratios of 2.3–2.9 for wood pellets, 1.7 for wood chips made of softwood and higher values for miscanthus and triticale pellets, which behave similarly to hardwoods. Alves et al., 2017 [169] reported that anhydrosugars represented 2.3–3.5 and 0.73–1.7 wt.% of the OC mass in emissions from the combustion of pellets and agrofuels, respectively, in a pellet stove. The authors came to the conclusion that the anhydrosugar mass fractions were 30 to 70 times lower than the amount reported for manually fired systems.

As noted by numerous studies, the levoglucosan-to-OC ratio has been shown to be highly variable in biomass burning emissions. Fine et al., 2001 [170] carried out tests to determine the chemical composition of fine PM emissions from the fireplace combustion of six species of wood grown in the northeastern United States. The authors found higher levoglucosan concentrations from hardwood than softwood biomass. Levoglucosan yields in the range of 0.109 to 0.168 g g^−1^ OC and 0.052 to 0.095 g g^−1^ OC were reported for hardwood and softwood combustion, respectively. On the other hand, softwood combustion usually produced higher mannosan emissions than those of hardwoods. Mannosan emissions ranged between 0.0013 and 0.0047 and between 0.0090 and 0.025 g g^−1^ OC for hardwood and softwood combustion, respectively. A later study [171,172,173] reported a levoglucosan-to-OC mass ratio of 0.136 g g^−1^ for fireplace combustion of four hardwood species grown in the southern US, which is consistent with the previous results. Furthermore, the researchers also proved the importance of combustion conditions to the anhydrosugars yield. After testing the same wood in a fireplace and in a woodstove, the authors reported that in general, the levoglucosan content in emissions from woodstoves were higher than those obtained from fireplaces. In addition to the L/M ratio, the relative proportion of levoglucosan to galactosan has also been used; in order to evaluate whether anhydrosugar ratios can provide information on the type of biofuel Caumo et al., 2016 [145], studied the levoglucosan-to-galactosan ratio (L/G), considering that hemicelluloses from sugarcane residue have a relatively high galactose (monosaccharide precursor of galactosan) content [17]. Comparing the results obtained in Brazil from an urban site with those obtained from a rural site characterized by sugarcane residue burning, it was found that a ratio value lower than 30 may be attributed to sugarcane burning at a regional scale and not to tropical forest fires affecting those areas.

### 4.2. Sugar Alcohols

Sugar alcohols, also referred to as *polyalcohols*, *polyols* or *sacharols*, represent another class of carbohydrate derivatives present in the atmospheric aerosol naturally produced by fungi, lichens, soil biota and algae [176]. Several studies have been undertaken to characterize sugar alcohols in different areas worldwide in order to use them as indicators of biogenic aerosol sources [131,177,178,179,180].

The reason why these sugars constituents are primary products of combustion is that they are formed either through direct volatilization from vegetation material or as products of the breakdown of polysaccharides. Some of these saccharide derivatives may also be formed by hydrolysis of the analogous anhydrosugars under the acidic atmospheric conditions produced by biomass burning, as suggested by many authors [55,56,57,58,161].

Arabitol and mannitol are two typical sugar alcohols widely monitored, as they make an important contribution to the mass of atmospheric aerosol particles derived from microbially degraded material during the leaf senescence period and from fungal spores [1,54,144,181,182,183]. In an atmospheric monitoring study in China, Kang et al., 2017, [181] found higher levels of mannitol and arabitol in spring likely, due to the blossoming of vegetation, and higher glycerol, arabitol and erythritol levels during winter and autumn, i.e., when vegetation decays and the fungal population increases. On the other hand, higher wintertime concentrations may be attributed to intense open burning of crop residues and indoor biofuel utilization for heating or cooking. Many other studies have suggested combustion sources of these compounds [57,58,133].

Zhang et al., 2013 [182] studied emissions from different types of biomass burning and proposed arabitol and inositol as useful tracers for green foliage combustion due to their higher concentrations of PM_2.5_ emitted from the combustion of leaves from a broadleaf shrub.

Similarly, in a study aimed at determining the emission factors of PM_2.5_ emitted from stoves by combustion of maize straw and wheat straw, Sun et al., 2019 [121] found different amounts of mannitol and inositol in such biofuels.

Schmidl et al., 2008 [142] found sugar alcohols in notable concentrations in leaf burning samples, suggesting the use of polyols to identify the contribution of leaf burning to high organic matter levels in ambient air.

Besides mannitol, inositol and arabitol, glycerol, xylitol and erythritol are also found in atmospheric monitoring studies (Table S1). In particular, erythritol, commonly present in soil microbial metabolites, is produced when soil combustion occurs following the burning of agricultural waste in fields [184]. It has also been widely detected during monitoring analysis, in addition to xylitol and sorbitol [60,127,128,133,185,186].

### 4.3. Primary Sugars

In addition to the saccharide compounds mentioned above, several studies have revealed that mono- and disaccharides are relatively abundant water-soluble organic components of atmospheric aerosols [187]. For example, glucose has been used as a marker for vegetable materials (such as leaves and pollen) and soil emissions in several studies [43,58], and trehalose is commonly associated with microbial and fungal activity [188,189].

Hence, biogenic sources of monosaccharides mainly include microorganism, vascular plants and animals [144], soil and associated biota [32].

Monosaccharides are thought to be relatively stable in the atmosphere [34], although studies focused on their atmospheric lifetime and spatial distribution are still limited. Nevertheless, atmospheric concentrations of monosaccharides have been quantified in several studies on PM emissions in both rural and urban areas. For example, Nirmalkar et al., 2015 [133], through an environmental monitoring study at a rural site in central India, found high concentrations of trehalose in PM_2.5_, attributing it to the thermal splitting of polysaccharides that occurs at high temperatures. Similarly, in a PM monitoring campaign in downtown Shanghai, Ren et al., 2020 [127] attributed the fraction composed of primary sugars (e.g., xylose, mannose, fructose, glucose, sucrose, maltose and trehalose, among others) to biomass burning. From this research emerged the possibility that anthropogenic emissions of primary sugars in the ambient air may occur by thermal stripping of cellulose during biomass burning events and be affected both by the type of fuel [190] and by the type of the combustion plant [191,192].

## 5. Conclusions

Publications worldwide agree on the identification of biomass burning as a major source of atmospheric particulate matter. The present work focused on literature findings concerning biomass burning emissions in order to provide better knowledge of saccharides tracers. As chemical and physical burning processes produce several compounds not attributable to other sources (e.g., fossil fuels), the detection of such specific markers is essential to evaluate the contribution of biomass burning to airborne particulate matter. A number of relevant mostly laboratory-based studies have contributed to our knowledge of the saccharide products of biomass combustion. Many of these studies have involved controlled pyrolysis of individual components or the burning of actual vegetation samples. Some characterization studies of smoke aerosols collected during field campaigns have also been carried out. By providing a picture of sugar compounds naturally and anthropologically emitted around the world, it is found that monosaccharide anhydrides, sugar alcohols and primary sugars have been widely reported as organic tracers for biomass burning. On the other hand, it has been demonstrated that emissions of such compounds depend not only on combustion characteristics and appliances but also on the type of biofuel and the atmospheric conditions. Hence, it is not currently possible to define a single compound as a universal marker of biomass combustion. In this field, in order to help researchers to obtain an accurate and realistic attribution of saccharide sources of ambient PM, future studies might be oriented toward the monitoring of specific saccharides in source apportionment studies and the integration of local emission information and dispersion models, as they have not yet been sufficiently investigated.

## Figures and Tables

**Table 1:** Source apportionment studies most used in the evaluation of saccharide compounds present in airborne particulate matter.

Compound	Source	SA Study	Reference
*Anhydrosugars*			
*Levoglucosan*	biomass burning	PMF	[43,123,124,125]
	biomass burning	PCA	[60,126,127,128]
*Mannosan*	biomass burning	PMF	[43]
	biomass burning	PCA	[60,126,127,128]
*Galactosan*	biomass burning	PMF	[43]
	biomass burning	PCA	[60,126,127,128]
***Sugar alcohols***			
*Inositol*	biomass burning	PCA	[58]
	fungal spores	PMF	[129]
	plants	PMF	
	soil dust		[130]
*Arabitol*	pollen	PMF	[131]
	yeasts, fungal spores	PMF	[43,132]
	fungal spores	PCA	[126]
	biomass burning	PCA	[58,133]
*Mannitol*	yeasts, fungal spores	PMF	[43,131,132]
	fungal spores	PCA	[126,134]
	biomass burning	PCA	[58,133]
	plants, soil	PCA	[128]
*Erythritol*	biomass burning	PMF	[43]
	biomass burning	PCA	[58,134]
	fungal spores	PCA	[134]
	plants, biota	PCA	[129,133]
*Glycerol*	biomass burning	PMF	[43,124]
	soil	PCA	[60]
	biomass burning	PCA	[58]
*Xylitol*	biomass burning	PCA	[58]
	plants, biota	PCA	[133]
	biomass burning	PMF	[125]
*Ribitol*	plants, biota	PCA	[128]
*Threitol*	biomass burning	PMF	[132]
	plants	PMF	[132]
*Methyltretol*	plants	PMF	[132]
***Monosaccharides***			
*Glucose*	pollen, pollen, fruits,	PMF	[131]
	fungal spores	PCA	[134]
	biomass burning	PCA	[58,60,128,133]
	soil, biota	PMF	[43,58]
*Fructose*	pollen fruits, plants	PMF	[131]
	fungal spores	PCA	[127,134]
	soil	PMF	[43]
	plants	PCA	[127]
*Galactose*	biomass burning	PMF	[43]
	biomass burning	PCA	[128]
*Arabinose*	biomass burning	PCA	[128]
*Mannopyranose*	soil, biota	PCA	[133]
*Xylose*	biomass burning	PCA	[60]
	plants	PCA	[129]
***Disaccharides***			
*Maltose*	biomass burning	PCA	[127]
	plants	PCA	[127]
*Sucrose*	plants, pollen, fruits	PMF	[131]
	plants	PCA	[127]
	pollen	PCA	[60,134]
	fungal spores	PCA	[129]
	soil, biota	PMF	[43]
*Trehalose*	yeasts, fungal spores	PCA	[127]
	soil biota	PCA	[58,133]
	biomass burning	PCA	[133]
	plants	PMF	[129]
	soil dust	PMF	[129,131]
	soil dust	PCA	[60]

**Table 2:** Saccharide compounds detected in aerosols emitted from tree and herbaceous biomass by means of different combustion appliances.

Biomass	Saccharides Detected														
Tree Biomass	Anhydrosugars			Sugar Alcohols					Mono- and Disaccharides				Units	Appliance	Ref.
*Hardwood*	L	M	G	I-ol	A-ol	M-ol	E-ol	S-ol	G-ose	F-ose	M-ose	S-ose			
Blue gum, Australian blackwood	13.4	7.59	5.2	-	-	-	-	-	-	-	-	74.6 (µg/g)	mg/g	Wildfire	[55]
Blue gum, Australian blackwood	12.8	5.65	2.8	-	-	-	-	-	-	-	-	-	mg/g	Wildfire	[55]
Acacia pellet	284	24.2	10.4	-	4.27	-	-	-	-	-	-	-	µg/g	Stove	[173]
Apple tree branch	3.44	0.22	0.18	<LOD	0.18	0.22	-	-	<LOD	-	-	0.08	mg/kg	Stove	[174]
Wood branch	5.46	0.36	0.29	<LOD	0.29	<LOD	-	-	<LOD	-	-	0.13	mg/kg	Stove	[121]
Pear and walnut wood and leaves	2.12	0.412	1.45	-	0.138	<0.005	-	0.23	-	<0.01	<0.01	<0.01	% of total mass	Open air combustion	[143]
Pear and walnut wood and leaves	2.14	0.36	1.93	-	0.14	0.008	-	0.27	-	<0.01	0.017	<0.02	% of total mass	Open air combustion	[143]
Red maple	213.16	11.06	3.97	-	-	-	-	-	-	-	-	-	mg/g	Stove	[131]
Sugar maple	210.07	12.88	2.55	-	-	-	-	-	-	-	-	-	mg/g	Stove	[131]
White oak	125.14	5.51	6.55	-	-	-	-	-	-	-	-	-	mg/g	Stove	[131]
*Softwood*															
Douglas fir	408.8	117.65	24.17	-	-	-	-	-	-	-	-	-	mg/g	Stove	[131]
Pine trees	13.9	8.4	6.21	-	-	-	-	-	-	-	-	-	mg/g	Wildfire	[162]
Pine trees	9.65	5.65	4.23	-	-	-	-	-	-	-	-	-	mg/g	Wildfire	[162]
Loblolly pine	253.11	46.33	11.45	-	-	-	-	-	-	-	-	-	mg/g	Stove	[131]
**Herbaceous biomass**															
Brooms, brambles	13.9	8.4	6.21	-	-	-	-	-	-	-	-	-	mg/g	wildfire	[162]
Rice straw	112	3.14	-	2.23	2.79	1.84	0.8	-	2.59	-	5.76	-	mg/kg	combustion chamber	[175]
Maize residues	33.5	1.27	-	1.7	1.59	1.23	0.59	-	1.14	-	2.22	-	mg/kg	combustion chamber	[175]
Leaf litter (ddf)	196	11	-	2.65	5.14	1.97	1.41	-	1.72	-	16.4	-	mg/kg	combustion chamber	[175]
Wheat straw	96.4	2.12	1.97	<LOD	0.21	0.11	-	-	0.56	-	-	<LOD	mg/kg	Stove	[174]
Wheat straw, corn straw	0.23	0.01	-	-	-	-	-	-	-	-	-	-	µg/m^3^	Stove	[77]
Wheat straw, corn straw	0.59	0.06	-	-	-	-	-	-	-	-	-	-	µg/m^3^	Stove	[77]

Abbreviation used: “L” = levoglucosan; “M” = mannosan; “G” = galactosan; “I-ol” = inositol; “A-ol” = arabitol; “M-ol” = mannitol; “E-ol” = erythritol; “S-ol” = sorbitol; “G-ose” = glucose; “F-ose” = fructose; “M-ose” = mannose; “S-ose” = sucrose; “LOD” = limit of detection; “-” = not detected or not specified.

## Data Availability

Data from this study are available from the corresponding author on reasonable request.

## Supplementary Materials

The following are available online at https://www.mdpi.com/article/10.3390/ijerph19074387/s1, Table S1: Saccharide compounds identified in the research articles reviewed [193,194,195,196,197].
