# Supplementary material for: Saccharides as Particulate Matter Tracers of Biomass Burning: A Review
Source: Int J Environ Res Public Health. 2022 Apr 6;19(7):4387. doi: 10.3390/ijerph19074387 (PMC8998709; doi:10.3390/ijerph19074387)
Supplement: Supplementary file 1 [file ijerph-19-04387-s001.zip › ijerph-1641222-supplementary.pdf]

International Journal of
Environmental Research and Public Health

MDPI

SACCHARIDES AS PARTICULATE MATTER TRACERS OF BIOMASS BURNING: A REVIEW

Table S1: Saccharide compounds identified in the research articles reviewed. Concentrations are expressed in ng/m^3.

Compounds	Location	Source	Fraction sampled	Period	Technique	Conc.	Reference
Levoglucosan							
	China	-	$PM_{2.5}$	Summer	GC-MS	89	[55]
	Thailand	-	$PM_{2.5}$	Winter-Spring	IC-PAD	1222	[57]
	Nepal	suburban background	$PM_{2.5}$	Spring	GC-MS	1230	[36]
	China	urban back-ground	$PM_{2.5}$	Autumn	GC-MS	110	[189]
	China	urban back-ground	$PM_{2.5}$	Winter	GC-MS	189	[189]
	China	urban back-ground	$PM_{2.5}$	Spring	GC-MS	63.4	[189]
	China	urban back-ground	$PM_{2.5}$	Summer	GC-MS	12.4	[189]
	China	rural back-ground	$PM_{2.5}$	Summer	HPAEC-PAD	205.94	[29]
	China	urban back-ground	$PM_{2.5}$	Summer	HPAEC-PAD	56.37	[29]
	USA	rural back-ground	$PM_{2.5}$	Autumn to Summer	GC-MS	38.4	[31]
	USA	urban background	$PM_{2.5}$	Winter to Spring	GC-MS	30.3	[31]
	India	urban background	PM_{10}	Spring	GC-MS	44.4	[190]
	India	rural background	PM_1	Autumn	GC-MS	1411	[54]
	China	urban background	$PM_{2.5}$	May-June	HPAEC-PAD	73	[191]
	China	urban background	$PM_{2.5}$	Nov-Dec	HPAEC-PAD	272	[191]
	China	suburban background	$PM_{2.5}$	May-June	HPAEC-PAD	8	[191]
	China	suburban background	$PM_{2.5}$	November	HPAEC-PAD	181	[191]
	India	rural background	$PM_{2.5}$	Autumn	IC-PAD	2258	[128]
	China	urban background	$PM_{2.5}$	Autumn	GC-MS	47.2	[122]
	China	urban background	$PM_{2.5}$	Winter	GC-MS	194.6	[122]
	China	urban background	$PM_{2.5}$	Spring	GC-MS	57.4	[122]
	China	urban background	$PM_{2.5}$	Summer	GC-MS	26.2	[122]
	China	urban background	$PM_{2.5}$	Spring	HPAEC-PAD	66	[130]
	China	urban background	$PM_{2.5}$	Summer	HPAEC-PAD	28	[130]

China	urban background	$PM_{2.5}$	Autumn	HPAEC-PAD	229	[130]
China	urban background	$PM_{2.5}$	Winter	HPAEC-PAD	161	[130]
Brazil	pasture site	aerosol	Autumn	GC-MS	2460	[53]
Brazil	forest site	aerosol	Autumn	GC-MS	1180	[53]
Italy	urban background	aerosol	Spring	IC-MS	-	[192]
Arctic region	rural background	PM_{10}	Spring	IC-MS	0.3	[193]
China	rural background	PM_{10}	Autumn to Winter	HPAEC-PAD	231.9	[127]
Serbia	urban background	aerosol	Summer to Winter	HPAEC-MS, HPLC-MS	424.9	[39]
Italy	urban site	$PM_{2.5}$	Summer	GC-MS	2.2	[123]
Italy	urban site	$PM_{2.5}$	Autumn	GC-MS	288.9	[123]
Italy	urban site	$PM_{2.5}$	Winter	GC-MS	259.4	[123]
Italy	urban site	$PM_{2.5}$	Spring	GC-MS	13.5	[123]
Italy	rural background	$PM_{2.5}$	Summer	GC-MS	<LOD	[123]
Italy	rural background	$PM_{2.5}$	Autumn	GC-MS	233.2	[123]
Italy	rural background	$PM_{2.5}$	Winter	GC-MS	252.9	[123]
Italy	rural background	$PM_{2.5}$	Spring	GC-MS	3.8	[123]
Greece	rural coastal site	PM_{10}	yearly	HPAEC-PAD	12.6	[194]
Brazil	forest site	$PM_{2.5}$	Winter	GC-MS	14.99	[195]
Brazil	forest site	PM_{10}	Winter	GC-MS	1.97	[195]
China	forest background	$PM_{2.5}$	Summer (daytime)	GC-MS	39	[56]
China	forest background	$PM_{2.5}$	Summer (nighttime)	GC-MS	5.3	[56]
Brazil	urban site	PM_{10}	Winter	GC-MS	260	[139]
Brazil	urban site	PM_{10}	Winter	GC-MS	400	[139]
Japan	marine site	aerosol	Winter	GC-MS	1.35	[178]
Japan	marine site	aerosol	Spring	GC-MS	0.79	[178]
Japan	marine site	aerosol	Summer	GC-MS	0.37	[178]
Japan	marine site	aerosol	Autumn	GC-MS	0.18	[178]
China	urban area	PM_1	Summer	HPAEC-PAD	14.7	[196]
China	urban area	$PM_{2.5}$	Summer	HPAEC-PAD	20.8	[196]
Taiwan	urban area	$PM_{2.5}$	Autumn	HPAEC-PAD	1.03	[177]
Czech Republic	suburban area	aerosol	Winter	NMR	-	[197]
Czech Republic	suburban area	aerosol	Summer	NMR	-	[197]

Mannosan							
	China	-	$PM_{2.5}$	Summer	GC-MS	4.4	[55]
	Thailand	-	$PM_{2.5}$	Winter-Spring	IC-PAD	65	[57]
	China	urban background	$PM_{2.5}$	Autumn	GC-MS	10.5	[189]
	China	urban background	$PM_{2.5}$	Winter	GC-MS	39.2	[189]
	China	urban background	$PM_{2.5}$	Spring	GC-MS	12.2	[189]
	China	urban background	$PM_{2.5}$	Summer	GC-MS	1.41	[189]
	China	rural background	$PM_{2.5}$	Summer	HPAEC-PAD	19.8	[29]
	China	urban background	$PM_{2.5}$	Summer	HPAEC-PAD	10.3	[29]
	India	urban background	PM_{10}	Spring	GC-MS	7.7	[190]
	India	rural background	PM_1	Autumn	GC-MS	19.1	[54]
	China	urban background	$PM_{2.5}$	May-June	HPAEC-PAD	6	[191]
	China	urban background	$PM_{2.5}$	November-December	HPAEC-PAD	17	[191]
	China	suburban background	$PM_{2.5}$	May-June	HPAEC-PAD	<LOD	[191]
	China	suburban background	$PM_{2.5}$	November	HPAEC-PAD	10	[191]
	China	urban background	$PM_{2.5}$	Autumn	GC-MS	3.8	[122]
	China	urban background	$PM_{2.5}$	Winter	GC-MS	15.3	[122]
	China	urban background	$PM_{2.5}$	Spring	GC-MS	3.9	[122]
	China	urban background	$PM_{2.5}$	Summer	GC-MS	2.3	[122]
	China	urban background	$PM_{2.5}$	Spring	HPAEC-PAD	15	[130]
	China	urban background	$PM_{2.5}$	Summer	HPAEC-PAD	12	[130]
	China	urban background	$PM_{2.5}$	Autumn	HPAEC-PAD	21	[130]
	China	urban background	$PM_{2.5}$	Winter	HPAEC-PAD	23	[130]
	Brazil	pasture site	aerosol	Autumn	GC-MS	126	[53]
	Brazil	forest site	aerosol	Autumn	GC-MS	49.5	[53]
	Italy	urban background	aerosol	Spring	IC-MS	-	[192]
	China	rural background	PM_{10}	Autumn to Winter	HPAEC-PAD	21.8	[127]
	Serbia	urban background	aerosol	Summer to Winter	HPAEC-MS, HPLC-MS	56.3	[39]
	Italy	urban site	$PM_{2.5}$	Summer	GC-MS	<LOD	[123]
	Italy	urban site	$PM_{2.5}$	Autumn	GC-MS	63.5	[123]

	Italy	urban site	$PM_{2.5}$	Winter	GC-MS	63.6	[123]
	Italy	urban site	$PM_{2.5}$	Spring	GC-MS	2	[123]
	Italy	rural background	$PM_{2.5}$	Summer	GC-MS	<LOD	[123]
	Italy	rural background	$PM_{2.5}$	Autumn	GC-MS	47	[123]
	Italy	rural background	$PM_{2.5}$	Winter	GC-MS	61.5	[123]
	Italy	rural background	$PM_{2.5}$	Spring	GC-MS	0.7	[123]
	Greece	rural coastal site	PM_{10}	yearly	HPAEC-PAD	1.53	[194]
	Brazil	forest site	$PM_{2.5}$	Winter	GC-MS	0.28	[195]
	Brazil	forest site	PM_{10}	Winter	GC-MS	0.08	[195]
	China	forest background	$PM_{2.5}$	Summer (daytime)	GC-MS	-	[56]
	China	forest background	$PM_{2.5}$	Summer (nighttime)	GC-MS	-	[56]
	Brazil	urban site	PM_{10}	Winter	GC-MS	19	[139]
	Brazil	urban site	PM_{10}	Winter	GC-MS	29.1	[139]
	Japan	marine site	aerosol	Winter	GC-MS	0.18	[178]
	Japan	marine site	aerosol	Spring	GC-MS	0.18	[178]
	Japan	marine site	aerosol	Summer	GC-MS	0.054	[178]
	Japan	marine site	aerosol	Autumn	GC-MS	0.055	[178]
	Taiwan	urban area	$PM_{2.5}$	Autumn	HPAEC-PAD	0.17	[177]
Galactosan							
	China	-	$PM_{2.5}$	Summer	GC-MS	10.6	[55]
	Thailand	-	$PM_{2.5}$	Winter-Spring	IC-PAD	14	[57]
	China	urban background	$PM_{2.5}$	Autumn	GC-MS	8.67	[189]
	China	urban background	$PM_{2.5}$	Winter	GC-MS	25.6	[189]
	China	urban background	$PM_{2.5}$	Spring	GC-MS	5.82	[189]
	China	urban background	$PM_{2.5}$	Summer	GC-MS	0.72	[189]
	China	rural background	$PM_{2.5}$	Summer	HPAEC-PAD	17.47	[29]
	China	urban background	$PM_{2.5}$	Summer	HPAEC-PAD	7.49	[29]
	India	urban background	PM_{10}	Spring	GC-MS	6.08	[190]
	India	rural background	PM_1	Autumn	GC-MS	5.52	[54]
	China	urban background	$PM_{2.5}$	Autumn	GC-MS	2.2	[122]
	China	urban background	$PM_{2.5}$	Winter	GC-MS	8.9	[122]

	China	urban background	$PM_{2.5}$	Spring	GC-MS	2.2	[122]
	China	urban background	$PM_{2.5}$	Summer	GC-MS	1	[122]
	Brazil	pasture site	aerosol	Autumn	GC-MS	55.4	[53]
	Brazil	forest site	aerosol	Autumn	GC-MS	22.7	[53]
	Italy	urban background	aerosol	Spring	IC-MS	-	[192]
	China	rural background	PM_{10}	Autumn to Winter	HPAEC-PAD	5.5	[127]
	Serbia	urban background	aerosol	Summer to Winter	HPAEC-MS, HPLC-MS	25.3	[39]
	Italy	urban site	$PM_{2.5}$	Summer	GC-MS	<LOD	[123]
	Italy	urban site	$PM_{2.5}$	Autumn	GC-MS	29.8	[123]
	Italy	urban site	$PM_{2.5}$	Winter	GC-MS	31.6	[123]
	Italy	urban site	$PM_{2.5}$	Spring	GC-MS	1.3	[123]
	Italy	rural background	$PM_{2.5}$	Summer	GC-MS	<LOD	[123]
	Italy	rural background	$PM_{2.5}$	Autumn	GC-MS	23.7	[123]
	Italy	rural background	$PM_{2.5}$	Winter	GC-MS	32	[123]
	Italy	rural background	$PM_{2.5}$	Spring	GC-MS	<LOD	[123]
	Greece	rural coastal site	PM_{10}	yearly	HPAEC-PAD	0.36	[194]
	Brazil	forest site	$PM_{2.5}$	Winter	GC-MS	0.19	[195]
	Brazil	forest site	PM_{10}	Winter	GC-MS	0.12	[195]
	China	forest background	$PM_{2.5}$	Summer (daytime)	GC-MS	-	[56]
	China	forest background	$PM_{2.5}$	Summer (nighttime)	GC-MS	-	[56]
	Brazil	urban site	PM_{10}	Winter	GC-MS	13.1	[139]
	Brazil	urban site	PM_{10}	Winter	GC-MS	16.7	[139]
	Japan	marine site	aerosol	Winter	GC-MS	0.054	[178]
	Japan	marine site	aerosol	Spring	GC-MS	0.036	[178]
	Japan	marine site	aerosol	summer	GC-MS	0.012	[178]
	Japan	marine site	aerosol	autumn	GC-MS	0.01	[178]
	Taiwan	urban area	$PM_{2.5}$	Autumn	HPAEC-PAD	1.83	[177]
Inositol (myo-inositol)							
	China	-	$PM_{2.5}$	Summer	GC-MS	1.7	[55]
	China	urban background	$PM_{2.5}$	Autumn	GC-MS	2.69	[189]
	China	urban background	$PM_{2.5}$	Winter	GC-MS	3.95	[189]
	China	urban background	$PM_{2.5}$	Spring	GC-MS	3.97	[189]

	China	urban back-ground	$PM_{2.5}$	Summer	GC-MS	1.29	[189]
	India	urban back-ground	PM_{10}	Spring	GC-MS	3.32	[190]
	India	rural back-ground	PM_1	Autumn	GC-MS	85.5	[54]
	India	rural back-ground	$PM_{2.5}$	Autumn	IC-PAD	170	[128]
	China	urban back-ground	$PM_{2.5}$	Spring	HPAEC-PAD	3.3	[130]
	China	urban back-ground	$PM_{2.5}$	Summer	HPAEC-PAD	2	[130]
	China	urban back-ground	$PM_{2.5}$	Autumn	HPAEC-PAD	6.9	[130]
	China	urban back-ground	$PM_{2.5}$	Winter	HPAEC-PAD	2.8	[130]
	Brazil	pasture site	aerosol	Autumn	GC-MS	2.9	[53]
	Brazil	forest site	aerosol	Autumn	GC-MS	1.8	[53]
	Brazil	forest site	$PM_{2.5}$	Winter	GC-MS	0.27	[195]
	Brazil	forest site	PM_{10}	Winter	GC-MS	2.85	[195]
	China	forest background	$PM_{2.5}$	Summer (daytime)	GC-MS	-	[56]
	China	forest back-ground	$PM_{2.5}$	Summer (nighttime)	GC-MS	-	[56]
	Japan	marine site	aerosol	Winter	GC-MS	0.03	[178]
	Japan	marine site	aerosol	Spring	GC-MS	0.06	[178]
	Japan	marine site	aerosol	Summer	GC-MS	0.06	[178]
	Japan	marine site	aerosol	Autumn	GC-MS	0.03	[178]
	Taiwan	urban area	$PM_{2.5}$	Autumn	HPAEC-PAD	3.43	[177]
Arabitol							
	Thailand	-	$PM_{2.5}$	Winter-Spring	IC-PAD	30	[57]
	China	urban back-ground	$PM_{2.5}$	Autumn	GC-MS	3.07	[189]
	China	urban back-ground	$PM_{2.5}$	Winter	GC-MS	3.34	[189]
	China	urban back-ground	$PM_{2.5}$	Spring	GC-MS	1.78	[189]
	China	urban back-ground	$PM_{2.5}$	Summer	GC-MS	0.46	[189]
	India	urban back-ground	PM_{10}	Spring	GC-MS	8.09	[190]
	India	rural back-ground	PM_1	Autumn	GC-MS	20.8	[54]
	India	rural back-ground	$PM_{2.5}$	Autumn	IC-PAD	174	[128]
	China	urban back-ground	$PM_{2.5}$	Autumn	GC-MS	3.9	[122]
	China	urban back-ground	$PM_{2.5}$	Winter	GC-MS	5.1	[122]
	China	urban back-ground	$PM_{2.5}$	Spring	GC-MS	2.9	[122]

	China	urban back-ground	$PM_{2.5}$	Summer	GC-MS	4	[122]
	USA	rural back-ground	$PM_{2.5}$	Autumn to Summer	GC-MS	3.1	[31]
	USA	urban background	$PM_{2.5}$	Winter to Spring	GC-MS	1.8	[31]
	China	urban background	$PM_{2.5}$	Spring	HPAEC-PAD	10	[130]
	China	urban back-ground	$PM_{2.5}$	Summer	HPAEC-PAD	6.9	[130]
	China	urban back-ground	$PM_{2.5}$	Autumn	HPAEC-PAD	11	[130]
	China	urban back-ground	$PM_{2.5}$	Winter	HPAEC-PAD	6.6	[130]
	Brazil	pasture site	aerosol	Autumn	GC-MS	19.5	[53]
	Brazil	forest site	aerosol	Autumn	GC-MS	19	[53]
	Italy	urban back-ground	aerosol	Spring	IC-MS	6	[192]
	Arctic region	rural back-ground	PM 10	Spring	IC-MS	0.8	[193]
	China	rural back-ground	PM_{10}	Autumn to Winter	HPAEC-PAD	11.7	[127]
	Serbia	urban back-ground	aerosol	Summer to Winter	HPAEC-MS, HPLC-MS	62.5	[39]
	Brazil	forest site	$PM_{2.5}$	Winter	GC-MS	13.84	[195]
	Brazil	forest site	PM_{10}	Winter	GC-MS	41.74	[195]
	China	forest background	$PM_{2.5}$	Summer (daytime)	GC-MS	-	[56]
	China	forest back-ground	$PM_{2.5}$	Summer (nighttime)	GC-MS	-	[56]
	Brazil	urban site	PM_{10}	Winter	GC-MS	9.3	[139]
	Brazil	urban site	PM_{10}	Winter	GC-MS	11.6	[139]
	Japan	marine site	aerosol	Winter	GC-MS	0.7	[178]
	Japan	marine site	aerosol	Spring	GC-MS	3.06	[178]
	Japan	marine site	aerosol	summer	GC-MS	3.08	[178]
	Japan	marine site	aerosol	autumn	GC-MS	1.68	[178]
	Czech Republic	suburban area	aerosol	Winter	NMR	-	[197]
	Czech Republic	suburban area	aerosol	Summer	NMR	-	[197]
Mannitol							
	Japan	marine site	aerosol	Winter	GC-MS	0.92	[178]
	Japan	marine site	aerosol	Spring	GC-MS	2.9	[178]
	Japan	marine site	aerosol	Summer	GC-MS	4	[178]
	Japan	marine site	aerosol	Autumn	GC-MS	2.07	[178]
	Thailand	-	$PM_{2.5}$	Winter-Spring	IC-PAD	12	[57]
	China	urban back-ground	$PM_{2.5}$	Autumn	GC-MS	3.28	[189]

China	urban background	$PM_{2.5}$	Winter	GC-MS	4.38	[189]
China	urban background	$PM_{2.5}$	Spring	GC-MS	4.86	[189]
China	urban background	$PM_{2.5}$	Summer	GC-MS	1.04	[189]
China	rural background	$PM_{2.5}$	Summer	HPAEC-PAD	61.3	[29]
China	urban background	$PM_{2.5}$	Summer	HPAEC-PAD	12.98	[29]
India	urban background	PM_{10}	Spring	GC-MS	10.3	[190]
India	rural background	PM_1	Autumn	GC-MS	968	[54]
India	rural background	$PM_{2.5}$	Autumn	IC-PAD	1623	[128]
China	urban background	$PM_{2.5}$	Autumn	GC-MS	15.7	[122]
China	urban background	$PM_{2.5}$	Winter	GC-MS	11.2	[122]
China	urban background	$PM_{2.5}$	Spring	GC-MS	8.5	[122]
China	urban background	$PM_{2.5}$	Summer	GC-MS	18.1	[122]
USA	rural background	$PM_{2.5}$	Autumn to Summer	GC-MS	3.8	[31]
USA	urban background	$PM_{2.5}$	Winter to Spring	GC-MS	3.2	[31]
China	urban background	$PM_{2.5}$	Spring	HPAEC-PAD	15	[130]
China	urban background	$PM_{2.5}$	Summer	HPAEC-PAD	21	[130]
China	urban background	$PM_{2.5}$	Autumn	HPAEC-PAD	22	[130]
China	urban background	$PM_{2.5}$	Winter	HPAEC-PAD	3.7	[130]
Brazil	pasture site	aerosol	Autumn	GC-MS	26.3	[53]
Brazil	forest site	aerosol	Autumn	GC-MS	22.3	[53]
Italy	urban background	aerosol	Spring	IC-MS	7	[192]
Arctic region	rural background	PM_{10}	Spring	IC-MS	0.7	[193]
China	rural background	PM_{10}	Autumn to Winter	HPAEC-PAD	23.3	[127]
Serbia	urban background	aerosol	Summer to Winter	HPAEC-MS, HPLC-MS	35.9	[39]
Italy	urban site	$PM_{2.5}$	Summer	GC-MS	3.1	[123]
Italy	urban site	$PM_{2.5}$	Autumn	GC-MS	3.4	[123]
Italy	urban site	$PM_{2.5}$	Winter	GC-MS	<LOD	[123]
Italy	urban site	$PM_{2.5}$	Spring	GC-MS	14.7	[123]
Italy	rural background	$PM_{2.5}$	Summer	GC-MS	27	[123]

	Italy	rural background	$PM_{2.5}$	Autumn	GC-MS	4.3	[123]
	Italy	rural background	$PM_{2.5}$	Winter	GC-MS	<LOD	[123]
	Italy	rural background	$PM_{2.5}$	Spring	GC-MS	6.3	[123]
	Greece	rural coastal site	PM_{10}	yearly	HPAEC-PAD	5.81	[194]
	Brazil	forest site	$PM_{2.5}$	Winter	GC-MS	15.17	[195]
	Brazil	forest site	PM_{10}	Winter	GC-MS	53.3	[195]
	China	forest background	$PM_{2.5}$	Summer (daytime)	GC-MS	-	[56]
	China	forest background	$PM_{2.5}$	Summer (nighttime)	GC-MS	-	[56]
	Brazil	urban site	PM_{10}	Winter	GC-MS	11.3	[139]
	Brazil	urban site	PM_{10}	Winter	GC-MS	14.8	[139]
	China	urban area	PM_1	Summer	HPAEC-PAD	9.8	[196]
	China	urban area	$PM_{2.5}$	Summer	HPAEC-PAD	15.2	[196]
	Czech Republic	suburban area	aerosol	Winter	NMR	-	[197]
	Czech Republic	suburban area	aerosol	Summer	NMR	-	[197]
Erythritol							
	Japan	-	aerosol	winter	GC-MS	0.21	[178]
	Japan	-	aerosol	Spring	GC-MS	0.62	[178]
	Japan	-	aerosol	Summer	GC-MS	1.22	[178]
	Japan	-	aerosol	Autumn	GC-MS	0.51	[178]
	China	-	$PM_{2.5}$	Summer	GC-MS	29.8	[55]
	Thailand	-	$PM_{2.5}$	Winter-Spring	IC-PAD	32	[57]
	China	urban background	$PM_{2.5}$	Autumn	GC-MS	4.64	[189]
	China	urban background	$PM_{2.5}$	Winter	GC-MS	6.13	[189]
	China	urban background	$PM_{2.5}$	Spring	GC-MS	1.69	[189]
	China	urban background	$PM_{2.5}$	Summer	GC-MS	0.52	[189]
	India	rural background	PM_1	Autumn	GC-MS	34.1	[54]
	India	rural background	$PM_{2.5}$	Autumn	IC-PAD	135	[128]
	China	urban background	$PM_{2.5}$	Autumn	GC-MS	1.3	[122]
	China	urban background	$PM_{2.5}$	Winter	GC-MS	2.6	[122]
	China	urban background	$PM_{2.5}$	Spring	GC-MS	1.2	[122]

	China	urban background	$PM_{2.5}$	Summer	GC-MS	2.1	[122]
	USA	rural background	$PM_{2.5}$	Autumn to Summer	GC-MS	0.6	[31]
	USA	urban background	$PM_{2.5}$	Winter to Spring	GC-MS	0.7	[31]
	China	urban background	$PM_{2.5}$	Spring	HPAEC-PAD	2.2	[130]
	China	urban background	$PM_{2.5}$	Summer	HPAEC-PAD	2.1	[130]
	China	urban background	$PM_{2.5}$	Autumn	HPAEC-PAD	4.7	[55]
	China	urban background	$PM_{2.5}$	Winter	HPAEC-PAD	2.9	[130]
	Brazil	pasture site	aerosol	Autumn	GC-MS	15.9	[53]
	Brazil	forest site	aerosol	Autumn	GC-MS	13.6	[53]
	Italy	urban background	aerosol	Spring	IC-MS	7	[192]
	Italy	urban site	$PM_{2.5}$	Summer	GC-MS	<LOD	[123]
	Italy	urban site	$PM_{2.5}$	Autumn	GC-MS	1.7	[123]
	Italy	urban site	$PM_{2.5}$	Winter	GC-MS	2.8	[123]
	Italy	urban site	$PM_{2.5}$	Spring	GC-MS	<LOD	[123]
	Italy	rural background	$PM_{2.5}$	Summer	GC-MS	<LOD	[123]
	Italy	rural background	$PM_{2.5}$	Autumn	GC-MS	<LOD	[123]
	Italy	rural background	$PM_{2.5}$	Winter	GC-MS	2.1	[123]
	Italy	rural background	$PM_{2.5}$	Spring	GC-MS	<LOD	[123]
	Brazil	forest site	$PM_{2.5}$	Winter	GC-MS	0.54	[195]
	Brazil	forest site	PM_{10}	Winter	GC-MS	1.84	[195]
	China	forest background	$PM_{2.5}$	Summer (daytime)	GC-MS	-	[56]
	China	forest background	$PM_{2.5}$	Summer (nighttime)	GC-MS	-	[56]
	Taiwan	urban area	$PM_{2.5}$	Autumn	HPAEC-PAD	0.14	[177]
Sorbitol							
	China	-	$PM_{2.5}$	Summer	GC-MS	5.5	[55]
	China	urban background	$PM_{2.5}$	Autumn	GC-MS	0.6	[122]
	China	urban background	$PM_{2.5}$	Winter	GC-MS	0.7	[122]
	China	urban background	$PM_{2.5}$	Spring	GC-MS	0.6	[122]
	China	urban background	$PM_{2.5}$	Summer	GC-MS	0.7	[122]
	USA	rural background	$PM_{2.5}$	Autumn to Summer	GC-MS	0.6	[31]
	USA	urban background	$PM_{2.5}$	Winter to Spring	GC-MS	0.4	[31]

	Brazil	pasture site	aerosol	Autumn	GC-MS	0.8	[53]
	Brazil	forest site	aerosol	Autumn	GC-MS	1.2	[53]
	Greece	rural coastal site	PM_{10}	yearly	HPAEC-PAD	0.72	[194]
	Czech Republic	suburban area	aerosol	Winter	NMR	-	[197]
	Czech Republic	suburban area	aerosol	Summer	NMR	-	[197]
Glycerol							
	China	-	$PM_{2.5}$	Summer	GC-MS	5.7	[55]
	China	urban back-ground	$PM_{2.5}$	Autumn	GC-MS	35.5	[189]
	China	urban back-ground	$PM_{2.5}$	Winter	GC-MS	35.6	[189]
	China	urban back-ground	$PM_{2.5}$	Spring	GC-MS	9.07	[189]
	China	urban back-ground	$PM_{2.5}$	Summer	GC-MS	5.84	[189]
	China	rural back-ground	$PM_{2.5}$	Summer	HPAEC-PAD	75.58	[29]
	China	urban back-ground	$PM_{2.5}$	Summer	HPAEC-PAD	46.97	[29]
	India	rural back-ground	PM_1	Autumn	GC-MS	32.7	[54]
	India	rural back-ground	$PM_{2.5}$	Autumn	IC-PAD	69.4	[128]
	China	urban back-ground	$PM_{2.5}$	Autumn	GC-MS	13.6	[122]
	China	urban back-ground	$PM_{2.5}$	Winter	GC-MS	22.3	[122]
	China	urban back-ground	$PM_{2.5}$	Spring	GC-MS	12.3	[122]
	China	urban back-ground	$PM_{2.5}$	Summer	GC-MS	11.2	[122]
	USA	rural back-ground	$PM_{2.5}$	Autumn to Summer	GC-MS	5.7	[31]
	USA	urban back-ground	$PM_{2.5}$	Winter to Spring	GC-MS	3.7	[31]
	Brazil	pasture site	aerosol	Autumn	GC-MS	8.2	[53]
	Brazil	forest site	aerosol	Autumn	GC-MS	5	[53]
	Brazil	forest site	$PM_{2.5}$	Winter	GC-MS	0.29	[195]
	Brazil	forest site	PM_{10}	Winter	GC-MS	2.06	[195]
	China	forest background	$PM_{2.5}$	Summer (daytime)	GC-MS	11.8	[56]
	China	forest background	$PM_{2.5}$	Summer (nighttime)	GC-MS	8.52	[56]
	China	forest back-ground	$PM_{2.5}$	Summer (daytime)	GC-MS	-	[56]
	China	forest back-ground	$PM_{2.5}$	Summer (nighttime)	GC-MS	-	[56]
	Taiwan	urban area	$PM_{2.5}$	Autumn	HPAEC-PAD	5.46	[177]

	Czech Republic	suburban area	aerosol	Winter	NMR	-	[197]
	Czech Republic	suburban area	aerosol	Summer	NMR	-	[197]
Xylitol							
	India	rural background	PM_1	Autumn	GC-MS	148	[54]
	India	rural background	$PM_{2.5}$	Autumn	IC-PAD	627	[128]
	China	urban background	$PM_{2.5}$	Autumn	GC-MS	0.2	[122]
	China	urban background	$PM_{2.5}$	Winter	GC-MS	0.3	[122]
	China	urban background	$PM_{2.5}$	Spring	GC-MS	0.2	[122]
	China	urban background	$PM_{2.5}$	Summer	GC-MS	0.2	[122]
	Greece	rural coastal site	PM_{10}	yearly	HPAEC-PAD	0.92	[194]
	Taiwan	urban area	$PM_{2.5}$	Autumn	HPAEC-PAD	3.7	[177]
Ribitol							
	USA	rural background	$PM_{2.5}$	Autumn to Summer	GC-MS	0.5	[31]
	USA	urban background	$PM_{2.5}$	Winter to Spring	GC-MS	0.1	[31]
	Italy	urban site	$PM_{2.5}$	Summer	GC-MS	4	[123]
	Italy	urban site	$PM_{2.5}$	Autumn	GC-MS	<LOD	[123]
	Italy	urban site	$PM_{2.5}$	Winter	GC-MS	<LOD	[123]
	Italy	urban site	$PM_{2.5}$	Spring	GC-MS	<LOD	[123]
	Italy	rural background	$PM_{2.5}$	Summer	GC-MS	4.3	[123]
	Italy	rural background	$PM_{2.5}$	Autumn	GC-MS	<LOD	[123]
	Italy	rural background	$PM_{2.5}$	Winter	GC-MS	<LOD	[123]
	Italy	rural background	$PM_{2.5}$	Spring	GC-MS	<LOD	[123]
Threitol							
	China	rural background	$PM_{2.5}$	Summer	HPAEC-PAD	10.47	[29]
	China	urban background	$PM_{2.5}$	Summer	HPAEC-PAD	6.12	[29]
	Brazil	pasture site	aerosol	Autumn	GC-MS	2.3	[53]
	Brazil	forest site	aerosol	Autumn	GC-MS	1.7	[53]
	China	rural background	PM_{10}	Autumn to Winter	HPAEC-PAD	8.4	[127]

Methyltetrols (2-methyl threitol)							
	China	rural background	$PM_{2.5}$	Summer	HPAEC-PAD	12.87	[29]
	China	urban background	$PM_{2.5}$	Summer	HPAEC-PAD	11.25	[29]
Glucose							
	Thailand	-	$PM_{2.5}$	Winter-Spring	IC-PAD	32	[57]
	China	urban background	$PM_{2.5}$	Autumn	GC-MS	5.02	[189]
	China	urban background	$PM_{2.5}$	Winter	GC-MS	16	[189]
	China	urban background	$PM_{2.5}$	Spring	GC-MS	19.5	[189]
	China	urban background	$PM_{2.5}$	Summer	GC-MS	2.84	[189]
	India	urban background	$PM_{2.5}$	Spring	GC-MS	13.77	[190]
	India	rural background	$PM_{2.5}$	Autumn	IC-PAD	2975	[128]
	China	urban background	$PM_{2.5}$	Autumn	GC-MS	4.8	[122]
	China	urban background	$PM_{2.5}$	Winter	GC-MS	3.6	[122]
	China	urban background	$PM_{2.5}$	Spring	GC-MS	3.4	[122]
	China	urban background	$PM_{2.5}$	Summer	GC-MS	2.8	[122]
	USA	rural background	$PM_{2.5}$	Autumn to Summer	GC-MS	10.2	[31]
	USA	urban background	$PM_{2.5}$	Winter to Spring	GC-MS	8	[31]
	Brazil	pasture site	aerosol	Autumn	GC-MS	36.7	[53]
	Brazil	forest site	aerosol	Autumn	GC-MS	20.1	[53]
	Italy	urban background	aerosol	Spring	IC-MS	21	[192]
	Serbia	urban background	aerosol	Summer to Winter	HPAEC-MS, HPLC-MS	21.6	[39]
	Italy	urban site	$PM_{2.5}$	Summer	GC-MS	0.9	[123]
	Italy	urban site	$PM_{2.5}$	Autumn	GC-MS	6.9	[123]
	Italy	urban site	$PM_{2.5}$	Winter	GC-MS	5.6	[123]
	Italy	urban site	$PM_{2.5}$	Spring	GC-MS	6.7	[123]
	Italy	rural background	$PM_{2.5}$	Summer	GC-MS	3.3	[123]
	Italy	rural background	$PM_{2.5}$	Autumn	GC-MS	4.9	[123]
	Italy	rural background	$PM_{2.5}$	Winter	GC-MS	5.4	[123]
	Italy	rural background	$PM_{2.5}$	Spring	GC-MS	7.3	[123]

	Greece	rural coastal site	PM_{10}	yearly	HPAEC-PAD	13.5	[194]
	Brazil	forest site	$PM_{2.5}$	Winter	GC-MS	7.86	[195]
	Brazil	forest site	PM_{10}	Winter	GC-MS	47.95	[195]
	China	forest background	$PM_{2.5}$	Summer (daytime)	GC-MS	16.2	[56]
	China	forest background	$PM_{2.5}$	Summer (nighttime)	GC-MS	4.79	[56]
	Brazil	urban site	PM_{10}	Winter	GC-MS	33	[139]
	Brazil	urban site	PM_{10}	Winter	GC-MS	31	[139]
	Japan	marine site	aerosol	Winter	GC-MS	1.99	[178]
	Japan	marine site	aerosol	Spring	GC-MS	5.47	[178]
	Japan	marine site	aerosol	Summer	GC-MS	5.6	[178]
	Japan	marine site	aerosol	Autumn	GC-MS	1.69	[178]
	China	urban area	PM_1	Summer	HPAEC-PAD	-	[196]
	China	urban area	$PM_{2.5}$	Summer	HPAEC-PAD	14.6	[196]
	Czech Republic	suburban area	aerosol	Winter	NMR	-	[197]
	Czech Republic	suburban area	aerosol	Summer	NMR	-	[197]
Fructose							
	China	-	$PM_{2.5}$	Summer	GC-MS	2.2	[55]
	China	urban background	$PM_{2.5}$	Autumn	GC-MS	2.18	[189]
	China	urban background	$PM_{2.5}$	Winter	GC-MS	6.18	[189]
	China	urban background	$PM_{2.5}$	Spring	GC-MS	3.19	[189]
	China	urban background	$PM_{2.5}$	Summer	GC-MS	1.58	[189]
	India	urban background	$PM_{2.5}$	Spring	GC-MS	19.43	[190]
	China	urban background	$PM_{2.5}$	Autumn	GC-MS	15.3	[122]
	China	urban background	$PM_{2.5}$	Winter	GC-MS	15.2	[122]
	China	urban background	$PM_{2.5}$	Spring	GC-MS	28.3	[122]
	China	urban background	$PM_{2.5}$	Summer	GC-MS	19.6	[122]
	China	urban background	$PM_{2.5}$	Spring	HPAEC-PAD	33	[130]
	China	urban background	$PM_{2.5}$	Summer	HPAEC-PAD	26	[130]
	China	urban background	$PM_{2.5}$	Autumn	HPAEC-PAD	20	[130]
	China	urban background	$PM_{2.5}$	Winter	HPAEC-PAD	8.2	[130]
	Brazil	pasture site	aerosol	Autumn	GC-MS	8.9	[53]
	Brazil	forest site	aerosol	Autumn	GC-MS	10.1	[53]

	Italy	urban background	aerosol	Spring	IC-MS	4	[192]
	Serbia	urban background	aerosol	Summer to Winter	HPAEC-MS, HPLC-MS	83.6	[39]
	Greece	rural coastal site	PM_{10}	yearly	HPAEC-PAD	10.4	[194]
	Brazil	forest site	$PM_{2.5}$	Winter	GC-MS	4.75	[195]
	Brazil	forest site	PM_{10}	Winter	GC-MS	28.7	[195]
	China	forest background	$PM_{2.5}$	Summer (daytime)	GC-MS	8.7	[56]
	China	forest background	$PM_{2.5}$	Summer (nighttime)	GC-MS	1.74	[56]
	Brazil	urban site	PM_{10}	Winter	GC-MS	27	[139]
	Brazil	urban site	PM_{10}	Winter	GC-MS	23	[139]
	Japan	marine site	aerosol	Winter	GC-MS	1.44	[178]
	Japan	marine site	aerosol	Spring	GC-MS	2.81	[178]
	Japan	marine site	aerosol	Summer	GC-MS	273	[178]
	Japan	marine site	aerosol	Autumn	GC-MS	0.62	[178]
	Czech Republic	suburban area	aerosol	Winter	NMR	-	[197]
	Czech Republic	suburban area	aerosol	Summer	NMR	-	[197]
Mannose							
	Thailand	-	$PM_{2.5}$	Winter-Spring	IC-PAD	33	[57]
	India	rural background	$PM_{2.5}$	Autumn	IC-PAD	290	[128]
	China	urban background	$PM_{2.5}$	Autumn	GC-MS	1.9	[122]
	China	urban background	$PM_{2.5}$	Winter	GC-MS	2.1	[122]
	China	urban background	$PM_{2.5}$	Spring	GC-MS	2	[122]
	China	urban background	$PM_{2.5}$	Summer	GC-MS	2.1	[122]
	China	urban background	$PM_{2.5}$	Spring	HPAEC-PAD	1	[130]
	China	urban background	$PM_{2.5}$	Summer	HPAEC-PAD	0.4	[130]
	China	urban background	$PM_{2.5}$	Autumn	HPAEC-PAD	0.8	[130]
	China	urban background	$PM_{2.5}$	Winter	HPAEC-PAD	-	[130]
	Brazil	pasture site	aerosol	Autumn	GC-MS	2.1	[53]
	Brazil	forest site	aerosol	Autumn	GC-MS	1.6	[53]
	Italy	urban background	aerosol	Spring	IC-MS	0.6	[192]
	Italy	urban site	$PM_{2.5}$	Summer	GC-MS	<LOD	[123]
	Italy	urban site	$PM_{2.5}$	Autumn	GC-MS	<LOD	[123]

	Italy	urban site	$PM_{2.5}$	Winter	GC-MS	<LOD	[123]
	Italy	urban site	$PM_{2.5}$	Spring	GC-MS	4	[123]
	Italy	rural background	$PM_{2.5}$	Summer	GC-MS	<LOD	[123]
	Italy	rural background	$PM_{2.5}$	Autumn	GC-MS	<LOD	[123]
	Italy	rural background	$PM_{2.5}$	Winter	GC-MS	<LOD	[123]
	Italy	rural background	$PM_{2.5}$	Spring	GC-MS	4	[123]
	Greece	rural coastal site	PM_{10}	yearly	HPAEC-PAD	1.71	[194]
Threalose							
	Japan	marine site	aerosol	winter	GC-MS	0.47	[178]
	Japan	marine site	aerosol	Spring	GC-MS	0.99	[178]
	Japan	marine site	aerosol	Summer	GC-MS	1.2	[178]
	Japan	marine site	aerosol	Autumn	GC-MS	0.27	[178]
	China	-	$PM_{2.5}$	Summer	GC-MS	3.9	[55]
	China	urban background	$PM_{2.5}$	Autumn	GC-MS	0.94	[189]
	China	urban background	$PM_{2.5}$	Winter	GC-MS	7.45	[189]
	China	urban background	$PM_{2.5}$	Spring	GC-MS	8.38	[189]
	China	urban background	$PM_{2.5}$	Summer	GC-MS	1.09	[189]
	India	urban background	$PM_{2.5}$	Spring	GC-MS	28.87	[190]
	India	rural background	$PM_{2.5}$	Autumn	IC-PAD	820	[128]
	China	urban background	$PM_{2.5}$	Autumn	GC-MS	2.4	[122]
	China	urban background	$PM_{2.5}$	Winter	GC-MS	2	[122]
	China	urban background	$PM_{2.5}$	Spring	GC-MS	2.4	[122]
	China	urban background	$PM_{2.5}$	Summer	GC-MS	3.5	[122]
	USA	rural background	$PM_{2.5}$	Autumn to Summer	GC-MS	0.9	[31]
	USA	urban background	$PM_{2.5}$	Winter to Spring	GC-MS	0.5	[31]
	Brazil	pasture site	aerosol	Autumn	GC-MS	8.9	[53]
	Brazil	forest site	aerosol	Autumn	GC-MS	8.7	[53]
	Brazil	forest site	$PM_{2.5}$	Winter	GC-MS	6.88	[195]
	Brazil	forest site	PM_{10}	Winter	GC-MS	48.07	[195]
	China	forest background	$PM_{2.5}$	Summer (daytime)	GC-MS	-	[56]
	China	forest background	$PM_{2.5}$	Summer (nighttime)	GC-MS	-	[56]

	Czech Republic	suburban area	aerosol	Winter	NMR	-	[197]
	Czech Republic	suburban area	aerosol	Summer	NMR	-	[197]
Galactose							
	India	rural background	$PM_{2.5}$	Autumn	IC-PAD	400	[128]
	Brazil	pasture site	aerosol	Autumn	GC-MS	0.9	[53]
	Brazil	forest site	aerosol	Autumn	GC-MS	0.6	[53]
	Serbia	urban background	aerosol	Summer to Winter	HPAEC-MS, HPLC-MS	8.6	[39]
	Italy	urban site	$PM_{2.5}$	Summer	GC-MS	<LOD	[123]
	Italy	urban site	$PM_{2.5}$	Autumn	GC-MS	2.8	[123]
	Italy	urban site	$PM_{2.5}$	Winter	GC-MS	3	[123]
	Italy	urban site	$PM_{2.5}$	Spring	GC-MS	1.2	[123]
	Italy	rural background	$PM_{2.5}$	Summer	GC-MS	<LOD	[123]
	Italy	rural background	$PM_{2.5}$	Autumn	GC-MS	2.6	[123]
	Italy	rural background	$PM_{2.5}$	Winter	GC-MS	3.1	[123]
	Italy	rural background	$PM_{2.5}$	Spring	GC-MS	<LOD	[123]
	Greece	rural coastal site	PM_{10}	yearly	HPAEC-PAD	0.91	[194]
	Taiwan	urban area	$PM_{2.5}$	Autumn	HPAEC-PAD	0.34	[177]
Arabinose							
	Italy	urban background	aerosol	Spring	IC-MS	0.4	[192]
	Arctic region	rural background	PM_{10}	Spring	IC-MS	0.7	[193]
	Italy	urban site	$PM_{2.5}$	Summer	GC-MS	<LOD	[123]
	Italy	urban site	$PM_{2.5}$	Autumn	GC-MS	2.4	[123]
	Italy	urban site	$PM_{2.5}$	Winter	GC-MS	9.5	[123]
	Italy	urban site	$PM_{2.5}$	Spring	GC-MS	<LOD	[123]
	Italy	rural background	$PM_{2.5}$	Summer	GC-MS	<LOD	[123]
	Italy	rural background	$PM_{2.5}$	Autumn	GC-MS	<LOD	[123]
	Italy	rural background	$PM_{2.5}$	Winter	GC-MS	8.9	[123]
	Italy	rural background	$PM_{2.5}$	Spring	GC-MS	<LOD	[123]
	Greece	rural coastal site	PM_{10}	yearly	HPAEC-PAD	1.37	[194]

Mannopyranose							
	India	rural background	$PM_{2.5}$	Autumn	IC-PAD	625	[128]
Xylose							
	China	urban background	$PM_{2.5}$	Autumn	GC-MS	8.96	[189]
	China	urban background	$PM_{2.5}$	Winter	GC-MS	10.2	[189]
	China	urban background	$PM_{2.5}$	Spring	GC-MS	2.34	[189]
	China	urban background	$PM_{2.5}$	Summer	GC-MS	0.78	[189]
	China	urban background	$PM_{2.5}$	Autumn	GC-MS	1.6	[122]
	China	urban background	$PM_{2.5}$	Winter	GC-MS	4.2	[122]
	China	urban background	$PM_{2.5}$	Spring	GC-MS	1.7	[122]
	China	urban background	$PM_{2.5}$	Summer	GC-MS	0.8	[122]
	Brazil	pasture site	aerosol	Autumn	GC-MS	12.7	[53]
	Brazil	forest site	aerosol	Autumn	GC-MS	7.3	[53]
	China	forest background	$PM_{2.5}$	Summer (daytime)	GC-MS	-	[56]
	China	forest background	$PM_{2.5}$	Summer (nighttime)	GC-MS	-	[56]
	Japan	marine site	aerosol	Winter	GC-MS	0.19	[178]
	Japan	marine site	aerosol	Spring	GC-MS	0.19	[178]
	Japan	marine site	aerosol	Summer	GC-MS	0.14	[178]
	Japan	marine site	aerosol	Autumn	GC-MS	0.06	[178]
	Czech Republic	suburban area	aerosol	Winter	NMR	-	[197]
	Czech Republic	suburban area	aerosol	Summer	NMR	-	[197]
Sucrose							
	China	-	$PM_{2.5}$	Summer	GC-MS	8.6	[55]
	China	urban background	$PM_{2.5}$	Autumn	GC-MS	0.11	[189]
	China	urban background	$PM_{2.5}$	Winter	GC-MS	1.65	[189]
	China	urban background	$PM_{2.5}$	Spring	GC-MS	0.47	[189]
	China	urban background	$PM_{2.5}$	Summer	GC-MS	0.34	[189]
	India	urban background	$PM_{2.5}$	Spring	GC-MS	75.24	[190]
	China	urban background	$PM_{2.5}$	Autumn	GC-MS	15	[122]
	China	urban background	$PM_{2.5}$	Winter	GC-MS	4.7	[122]

	China	urban background	$PM_{2.5}$	Spring	GC-MS	18.5	[122]
	China	urban background	$PM_{2.5}$	Summer	GC-MS	29.8	[122]
	USA	rural background	$PM_{2.5}$	Autumn to Summer	GC-MS	0.9	[31]
	USA	urban background	$PM_{2.5}$	Winter to Spring	GC-MS	0.5	[31]
	Brazil	pasture site	aerosol	Autumn	GC-MS	7.2	[53]
	Brazil	forest site	aerosol	Autumn	GC-MS	14.6	[53]
	Italy	urban background	aerosol	Spring	IC-MS	25	[192]
	Serbia	urban background	aerosol	Summer to Winter	HPAEC-MS, HPLC-MS	10.8	[39]
	Italy	urban site	$PM_{2.5}$	Summer	GC-MS	<LOD	[123]
	Italy	urban site	$PM_{2.5}$	Autumn	GC-MS	4.6	[123]
	Italy	urban site	$PM_{2.5}$	Winter	GC-MS	3.1	[123]
	Italy	urban site	$PM_{2.5}$	Spring	GC-MS	1.7	[123]
	Italy	rural background	$PM_{2.5}$	Summer	GC-MS	<LOD	[123]
	Italy	rural background	$PM_{2.5}$	Autumn	GC-MS	6.2	[123]
	Italy	rural background	$PM_{2.5}$	Winter	GC-MS	2.6	[123]
	Italy	rural background	$PM_{2.5}$	Spring	GC-MS	<LOD	[123]
	Greece	rural coastal site	PM_{10}	yearly	HPAEC-PAD	8.44	[194]
	Brazil	forest site	$PM_{2.5}$	Winter	GC-MS	1.45	[195]
	Brazil	forest site	PM_{10}	Winter	GC-MS	36.95	[195]
	China	forest background	$PM_{2.5}$	Summer (daytime)	GC-MS	-	[56]
	China	forest background	$PM_{2.5}$	Summer (nighttime)	GC-MS	-	[56]
	Japan	marine site	aerosol	Winter	GC-MS	2.67	[178]
	Japan	marine site	aerosol	Spring	GC-MS	1.66	[178]
	Japan	marine site	aerosol	Summer	GC-MS	0.89	[178]
	Japan	marine site	aerosol	Autumn	GC-MS	0.24	[178]
	Czech Republic	suburban area	aerosol	Winter	NMR	-	[197]
	Czech Republic	suburban area	aerosol	Summer	NMR	-	[197]
Maltose							
	China	urban background	$PM_{2.5}$	Autumn	GC-MS	1	[122]
	China	urban background	$PM_{2.5}$	Winter	GC-MS	1.6	[122]
	China	urban background	$PM_{2.5}$	Spring	GC-MS	1.3	[122]

	China	urban background	$PM_{2.5}$	Summer	GC-MS	1.6	[122]
	China	forest background	$PM_{2.5}$	Summer (daytime)	GC-MS	-	[56]
	China	forest background	$PM_{2.5}$	Summer (nighttime)	GC-MS	-	[56]
Glucopyranose							
	China	-	$PM_{2.5}$	Summer	GC-MS	11.8	[55]

Note: "LOD" = limit of detection; " - "= not available.
